# Epidemics from a Chemical Standpoint*From Bulletin of the Medico-Legal Society of New York, January, 1879.

**Published:** 1879-11

**Authors:** R. Ogden Doremus

**Affiliations:** Professor of Chemistry and Toxicology in Bellevue Hospital Medical College; Professor of Chemistry and Physics in the College of the City of New York; Chemist of the Medico-Legal Society


EPIDEMICS FROM A CHEMICAL STANDPOINT.* By R. OGDEN DOREMUS, M.D., LL.D., Professor of Chemistry and Toxicology in Bellevue Hospital Medical College; Professor of Chemistry and Physics in the College of the City of New York; Chemist of the Medico-Legal Society.

Dr. C. R. Agnew, formerly attached to the New York Hospital, informs me that in consequence of the reception of patients suffering from "ship fever," the north wing of the old building near Duane street became so saturated with disease-breeding agents that it had to be abandoned. This was about twenty-five years ago. The patients were removed from this wing; the windows were kept open for many weeks to accomplish thorough ventilation; the walls and ceilings of the wards were scraped and whitewashed, but in vain! Even the workmen engaged in this cleansing process were taken sick, and one or more died.

* From Bulletin of the Medico-Legal Society of New York, January, 1879.

So many hospitals, or parts of hospitals, in Europe and in this country have become magazines of disease, puerperal fevers, pyæmia, etc., that many savans urge that the buildings shall be constructed of *wood*, and be burned up every second or third year.

When Pandora, the all-gifted, opened the fabled box and released its pernicious contents, which brought immeasurable suffering upon mankind, "the terrified female at length gained sufficient presence of mind to close the lid; and Hope thereupon was alone secured."

Prometheus, who formed the first man out of clay, kindled his torch from the chariot of Phœbus, with which he blew eternal flames into the breasts of his creatures. Notwithstanding the ire of Jupiter, he ascended a second time and brought fire from the sun.

The chemist was anciently designated the "philosopher by fire." Perhaps we are justified in saying that to him we should look to antidote, at least, certain of the evils set free by her whom Vulcan made, and intended to be the consort of the son of Iapetus.

By the potency of modern science, Hope has been released from Pandora's box.

We propose to show that spirit can be set at war with spirit, the combat resulting favorably to humanity.

There are no firmer believers in spirits than chemists; for they can make them of every hue of the rainbow, or colorless and invisible, or even black; of varying weights and odors; some grateful to our senses, others nauseating and disgusting; markedly differing in chemical qualifications — one the spirit of life, the vital air; while a host "whose name is legion" exist, and can at will be produced, which are destructive to life.

With but few exceptions, all poisonous emanations from humanity, in health or in disease, are compounds of the most gaseous of all gases, hydrogen.

This lightest of all known elements, when just released from combination with others, or in its "nascent" state, possesses marvellous powers of affinity. Thus, one pound of hydrogen can unite with sixteen pounds of sulphur, and can confer on this solid body its own condition of gaseity. With carbon it yields forms of illuminating gas; with many metals, as iron, zinc, arsenic, antimony, etc., it produces spirit-like bodies, some of which are most poisonous. Such substances as pus, vaccine virus, etc., are compounds of hydrogen; and future chemists will doubtless discover that all disease-propagating gases or germs consist in part of this particular ghost.

If, therefore, we can dehydrogenate them, or rob them of this spirit, we can destroy their power for evil.

To employ a political figure, chlorine gas is the great "ring breaker." It seizes upon the chief of the ring, *Hydrogen*, imprisons it, and breaks up the evil combination.

I have here a tall glass jar, the upper part of which contains sulphuretted hydrogen gas, the lower part water. On bubbling up through the water, this greenish-yellow gas, chlorine, as it reaches the ill-odored gas above, instantly deprives it of its hydrogen (forming with it hydrochloric acid); and you observe the yellow deposit of sulphur on the water, and upon the sides of the jar.

I now present a compound of carbon and hydrogen to this chlorine — black carbon is at once deposited, the hydrogen, by preference, combining with the chlorine.

Should I introduce the most poisonous form of arsenic, viz., arseniuretted hydrogen, to chlorine, the superior affinity of hydrogen for chlorine would be immediately pronounced; the hydrogen and chlorine would unite as before, and should there be an excess of chlorine, a chloride of arsenic would be formed.

Should chlorine be exhibited to a mixture of sulphuretted hydrogen and arseniuretted hydrogen gasses, it would deprive them both of their hydrogen; the sulphur and the arsenic, being in a "nascent" state, would unite, and form a sulphide of arsenic, yellow orpiment.

If a glass rod be dipped in anhydrous hydrocyanic (prussic) acid, which has been liquified by cold, on approaching the rod with its adherent drop to a rabbit, the animal falls dead before the poisonous liquid touches him — due to the inhalation of the vaporous or gaseous acid. If chlorine be presented to this most rapidly fatal of all the spirits, it instantly disarms it of its virulence by robbing it of its hydrogen.

If equal volumes of hydrogen and chlorine be placed in a glass flask in a dark room, on exposing them to the sunbeam they combine with explosive violence.

Hence we feel warranted in the assertion that if chlorine gas can be generated in great volume, and especially with the aid of moisture, which facilitates its action, it can dehydrogenate any of the virulent spirits which result from vegetable and animal decomposition.

In 1849 I was consulted by one of our largest transatlantic steamship companies as to the best method of disinfecting one of the state-rooms, which had been occupied by a passenger in whom small-pox developed a few days after leaving Liverpool.

The liberal generation of chlorine gas in said compartment accomplished the desired result.

In the summer of 1865 I had the pleasure of meeting, on Broadway, Prof. Lewis A. Sayre, M.D., then Health Physician to our city. He announced that the steamship *Atlanta* had arrived at quarantine from Liverpool with a large number of steerage passengers; that sixty of them had died of cholera! He requested me to accompany him to the Mayor's office to discuss what should be done.

I expressed to Prof. Sayre and Mayor Gunther that chemical agents, *if liberally employed*, could decompose and destroy all these disease-spreading agents; that ships could be disinfected in twenty-four or forty-eight hours better than by a detention of forty days for ventilation; that modern chemistry would enable us to almost abolish *quarantine* by shortening the customary delay which its name involves.

*Carte blanche* was given by the Mayor.

Accompanied by my assistant, Dr. A. W. Wilkinson, a visit was paid to Dr. Swinburne, then Health Officer at quarantine, who gave his cordial co-operation.

Among the most efficient agents with which we successfully disinfected the ship referred to as freighted with disease, and many other vessels which subsequently arrived from dangerous ports, was chlorine gas. Rolls of sheet lead were taken on board the vessels, with hundreds of pounds of common salt and peroxide of manganese; also, carboys of sulphuric acid. The lead was unrolled, turned up at the edges, so as to make receptacles eight or ten feet in length and four feet in breadth. Into these troughs the salt and the manganese were mingled with water, forming a black mud, and when all were ready to leave the hold or part of the vessel where the troughs had been placed, a carboy or more of sulphuric acid was poured into the aforesaid mixture.

Huge volumes of chlorine were immediately given off; hundreds of pounds of this greenish-colored gas arose and diffused in every direction. The hatches were "battened down," and this potent element was left to accomplish our purpose, viz., to dehydrogenate, and thus decompose, the death-breeding gases and germs.

That this mode of treatment was eminently successful, was demonstrated by the fact that not a single case of cholera appeared in this city, or the cities connected with this port, or the section of our country to which the emigrants passed. So complete was the disinfection that not only were the ships purified, but also the articles of merchandise from infected ports, as well as the baggage of cabin passengers and emigrants. In previous years cholora was known to have been developed in the interior of this State from boxes and trunks containing household articles which had been brought from parts of Europe where the disease existed.

Who can estimate the loss of life and property had cholera been introduced into New York, and thus probably have been disseminated through the country?

I am happy to learn that this process has been continued by my quondam college-mate, Dr. S. Oakley Vanderpoel, the present Health Officer at Quarantine, to whom our city and the country is indebted for his faithful and efficient services.

Three years ago I was asked by the present Commissioners of Charities and Correction to undertake the purification and disinfection of the surgical wards of Bellevue Hospital. In certain wards pyæmia had proved very fatal. The *heroic* chlorine treatment (if I may use this expression) was adopted for the wards, and ozone for the water closets.

Strips of paper were pasted over the crevices around the windows and doors. Troughs of lead, as before described, were brought into the ward; a sack of salt was emptied into them, and about an equal weight of black oxide of manganese. These were intimately mixed, by stirring them with water, by means of wooden shovels. A carboy of sulphuric acid was emptied into pitchers, basins and other vessels, and placed by the sides of the troughs. The floors were then wetted with water, and steam was allowed to escape from the heaters until the condensed moisture had dampened the ceilings and was trickling down the walls.

Half a dozen assistants groped their way with me through the mist to the sides of the most remote trough; each of us simultaneously poured out the contents of the vessels filled with sulphuric acid upon the salt and manganese, repeating this operation at the second trough. We then hastily made our exit, and nailed up the door, lest any one should accidentally enter; for the amount of chlorine thus liberated would have proved fatal to any one who might have ventured into its presence.

The next day the windows were opened from the outside, and after an hour or more of ventilation, we entered, and having first filled the earthen vessels with sulphuric acid, we stirred up the mass resulting from the previous day's reaction, then added this second dose of acid, and again rapidly retreated and secured the door.

In the first ward, where pyæmia had proved most fatal, the contents of the troughs, chiefly sulphate of soda and sulphate of manganese, were removed, and a second sack of salt, with its equivalent of peroxide of manganese, were mixed in them with water, and the full complement of sulphuric acid poured into the mixture, after filling the compartment with steam as before.

The walls and floors were then washed and scrubbed, after a third liberal treatment with chlorine was completed, twenty-four hours having been allotted for each dose of chlorine to fulfill its fell purpose, viz., to permeate the *plaster walls*, and the *very stones* of the hospital in search of its prey, the foul, death-dealing gases and germs that lurked in these hiding places defying all ordinary methods of removal.

After a day's ventilation and drying, the disinfection was considered complete. It was a pleasure to pass from the adjoining wards into the purified one; the mawkish flavor, so common to even the most cleanly hospitals, had entirely disappeared.

To test the efficiency of the chlorine gas, the cotton sheets were left on some of the beds when the first charge was employed. The next day these sheets were so tender that the least touch sufficed to crumble them to pieces. The woolen blankets were disinfected, but uninjured.

All the other surgical wards in the north wing of Bellevue Hospital were treated twice with the most liberal charge of chlorine. Estimating from the quantities of the chemicals employed, between *two* and *three tons* of chlorine gas must have been produced by the reaction!

The method described for generating chlorine is preferable to the employment of hydrochloric (muriatic) acid and the binoxide of manganese, because of the more copious yield of gas, as may be shown by the following formulæ:

$$2(SO_3\ H_2O) + MnO_2 + Na\ Cl = SO_3MnO + SO_3NaO + 2(H_2O) + Cl.$$

Here all the chlorine is liberated from the common salt (chloride sodium), the sulphate of manganese and the sulphate of soda remaining; whereas, by the use of hydrochloric acid, only one-half of the chlorine is discharged as a gas; thus,

$$4\ (H\ Cl) + MnO_2 = Mn\ Cl_2 + 2\ (H_2O) + Cl_2,$$

the other portion combining with the manganese to form the chloride of this metal.

The first process generates heat by the action of the sulphuric acid and the water. This facilitates the diffusion of the heavy gas.

In consequence of the very pronounced odor of chlorine gas many persons, including physicians, and even those who have written on the subject of its applicability as a disinfectant, err in regard to the *quantity* that should be relied on. One distinguished physician narrated to me that he signally failed. He placed some manganese in a *saucer* and poured hydrochloric acid upon it! Need we wonder at the result?

In a recent medical journal sent to me, a description of modes of disinfection is given; under the head of chlorine the writer says, "Place four ounces of manganese on a plate and add hydrochloric acid to it!"

There are incidental advantages in resorting to sheets of lead as a suitable receptacle for the chemicals. Troughs of any length can be easily and quickly made; and after the residues of the reactions have been removed they can be rolled up into a compact form. By unrolling them and turning up the edges they can be used again, and as often as desired. The sulphuric acid forms an insoluble sulphate of lead on the inner surface, so that the metal is not perforated or destroyed.

The water-closets connected with the surgical wards of Bellevue Hospital were deodorized and disinfected by sprinkling in the urinals, for a number of consecutive nights a mixture of the manganate of soda and the sulphate of magnesia.

In contact with water these salts produce the *permanganate* of soda, and at little cost. The reaction is thus expressed by symbols:

$$3(Mn\,O_3\,Na_2O)+2(SO_3\,MgO=$$

$$(Mn_2\,O_7\,Na_2O)+MnO_2+2(SO_3\,Na_2O)+2(MgO).$$

Permanganate Soda + Binox: Manganese + 2(Sulphate Soda) + 2(Magnesia.)

In contact with the impurities of the urinals the permanganate of soda decomposes, yielding a most active form of oxygen, nascent oxygen,or ozone, which at once deodorizes and disinfects.

This mixture has no unpleasant flavor such as characterizes carbolic acid, cresylic acid, chloride of lime, hypochlorite of soda (Labarraque's solution), and many other disinfectants. It may be constantly applied without annoying the patients of the sick room or hospital. It should be kept by the side of the urinal, and the mixed powders should be thrown into the vase or urinal each time it is used.

The liberal generation of chlorine gas has been repeated at Bellevue every few months, and with the happiest results. The most sanguine anticipations could not surpass the statement made by Dr. James R. Wood, the oldest surgeon of the hospital, that "since this thorough purification no case of pyæmia has originated in the surgical wards!"

A few years ago the Commissioners of Charities and Correction had under discussion the advisability of stripping the walls of Bellevue and relining the edifice with wood and plaster. I doubt if even this expensive procedure would have accomplished a much better result than was obtained by scraping the walls of the old New York Hospital, for the very stones were doubtless impregnated with poison.

If we ignore chemistry, the ancient Hebraic process is the only efficient one — to "break down the house, the stones of it, and the timber thereof, and all the mortar of the house, and carry them forth out of the city into an unclean place;" unless we construct our hospitals of *wood*, and burn them as occasion demands.

The latter method would effectually carry out the signification of the word *purify* — the treatment by fire.

But in a large city we need hospitals distributed within its precincts. The application of the torch might accomplish more destruction than anticipated, unless hospitals were in the outskirts of our cities. There are seventeen hospitals in different parts of Paris.

To transport patients several miles diminishes their chances of recovery, especially "accident cases."

The explosion of the Staten Island ferry-boat *Westfield* at the South Ferry, shortly after the demolition of the old New York Hospital, demonstrated the necessity of a commodious hospital in the lower part of the city. Many of the scalded and mutilated victims of this horrible accident had to be conveyed a distance of three miles to Bellevue!

From 10 a.m. until three o'clock in the afternoon the major part of the active brains of New York is in the lower extremity of our city. Accidents may occur to the rich man as well as to his hired laborer. Some of our wealthiest men have died in their down-town offices. Four years ago one of the mayors of New York died in the City Hall. Medical men of distinction were miles away. Had there been a hospital down town, physicians of experience could have been commanded instantly.

Some may reply, we have provided a few beds in the lower part of the city where the sick and injured may be temporarily cared for. But those in attendance are mostly young men, and although of undoubted ability, have not enjoyed the years of practical knowledge which might be preferred.

If a large hospital was established, say on the Battery, the most eminent surgeons and physicians of our city would be competitors for the important positions, and thus at all times, assuredly of the day, their services might be commanded. The attention of our citizens, and of the Commissioners of Charities and Corrections, should be called to consider the advisability of some such provision.

One of the members of the Board of Trustees of the New York Hospital, distinguished for his deep interest in caring for the sick, and whose liberality has recently been expressed in the erection of a surgical pavilion at Bellevue, favored me with a visit, at my office, shortly after the completion of the elegant new hospital in Fifteenth street, between Fifth and Sixth Avenues. The purpose of the visit was to solicit the details of the chemical treatment in the purification of Bellevue Hospital.

He stated that attacks had been made in the public journals upon the folly and extravagance of the Board in constructing the elaborate and expensive building, which at any time, and even within a few months, might become in part impregnated with disease, so as to be unfitted for the reception and treatment of patients; and like the north wing of the old New York Hospital in Broadway (just above the City Hall Park), would have to be abandoned, and this in the face of the views of many European and American physicians, that hospitals should be of wood, and burned every second and third year.

We feel justified in claiming, as the result of personal experience, that the ancient destructive methods may be abolished; that by resorting to the active and diffusive chemical agents, in *sufficient quantities*, not only may clothing and goods be thoroughly disinfected, but that ships, and even houses and hospitals of solid masonry, may be speedily and completely purified.

Chemistry, *heroically* employed, may save nations from the miscalled "visitations of Providence," which are really resultants of *improvidence*, ignorance and neglect. Thousands of lives may be spared; innumerable sufferings avoided; the retardation of commerce, by the forty days' delay at our ports, prevented, which involves millions of money; our dwellings and commodious hospitals kept in a fitting condition for those enjoying the blessings of health or suffering from accident or disease. —*Sanitarian*, July, 1879.

---

TREATMENT OF CHOREA. (J. Simon, *Gaz. Obstet.*, Sept. 5, 1879.)

The patient had been treated with syrup of iodide of iron, bromide of zinc, saline and sulphur baths. This treatment is highly rational. There is no specific for chorea. One may calm the disordered movements and prevent the accidents from increasing, but cannot arrest this affection. Cases have been seen suspended in eight days, while others, in spite of us, continue six weeks, six months, six years. They are then dependent upon a cerebral lesion. Chorea may be said to have a natural course of three months. At this time it disappears of itself. However, something must be done. The patient (5 years old) was ordered syrup of chloral, a teaspoonful three times a day, in a little syrup of chamomile, salt baths, wine of cinchona before each meal.

When the physician is called at the outset of the disease, dry cups should be applied along the spine each morning. In the way of calmants, he gives tinctures of aconite or hemlock, also tincture of colchicum as an antirheumatic. The acute stage being past, he orders tonics, posphate of lime, saline baths, gymnastics. He does not advise the seaside for excitable subjects. If one desires the vivifying effect of the sea air, it is best to keep the little patients at some distance from the shore.

MR. J. KNOWSLEY THORNTON, in a recent letter to Dr. C. T. Parkes, of this city, reports that Mr. Keith, of Edinburgh, has performed 60 ovariotomies and 5 hysterotomies, without a single fatal result.

Mr. Keith is a well-known advocate of the Lister system, and carries out its rules in detail with the most careful attention.

